# Characterization of glycerol-3-phosphate acyltransferase 9 (*AhGPAT9*) genes, their allelic polymorphism and association with oil content in peanut (*Arachis hypogaea* L.)

**DOI:** 10.1038/s41598-020-71578-7

**Published:** 2020-09-04

**Authors:** Yuying Lv, Xiurong Zhang, Lu Luo, Hui Yang, Pinghua Li, Kun Zhang, Fengzhen Liu, Yongshan Wan

**Affiliations:** State Key Laboratory of Crop Biology, Shandong Key Laboratory of Crop Biology, College of Agronomy, Shandong Agricultural University, Tai’an, 271018 Shandong China

**Keywords:** Natural variation in plants, Natural variation in plants, Natural variation in plants, Natural variation in plants, Natural variation in plants

## Abstract

GPAT, the rate-limiting enzyme in triacylglycerol (TAG) synthesis, plays an important role in seed oil accumulation. In this study, two *AhGPAT9* genes were individually cloned from the A- and B- genomes of peanut, which shared a similarity of 95.65%, with 165 site differences. The overexpression of *AhGPAT9* or the knock-down of its gene expression increased or decreased the seed oil content, respectively. Allelic polymorphism analysis was conducted in 171 peanut germplasm, and 118 polymorphic sites in *AhGPAT9A* formed 64 haplotypes (*a1* to *a64*), while 94 polymorphic sites in *AhGPAT9B* formed 75 haplotypes (*b1* to *b75*). The haplotype analysis showed that *a5*, *b57*, *b30* and *b35* were elite haplotypes related to high oil content, whereas *a7*, *a14*, *a48*, *b51* and *b54* were low oil content types. Additionally, haplotype combinations *a62*/*b10*, *a38*/*b31* and *a43*/*b36* were associated with high oil content, but *a9*/*b42* was a low oil content haplotype combination. The results will provide valuable clues for breeding new lines with higher seed oil content using hybrid polymerization of high-oil alleles of *AhGPAT9A* and *AhGPAT9B* genes.

## Introduction

Plant lipids, including glycerolipids, membrane lipids, signaling molecules, photosynthetic pigments, plant hormones and plant surface protective substances, play important roles in plant growth, development and stress responses. Glycerolipids, including phospholipids, glycolipids, triacylglycerol, and extracellular lipids such as cutin and suberin, are the main components of plant lipids, and are formed by the acylation of glycerol at the sn-1, sn-2, or sn-3 sites using glycerol as the molecular framework^1^. Triacylglycerol (TAG) is the main form of plant storage oil, and accumulates in the flower petals, pollen grains, developing seeds and fruits of many plant species^2,3^, providing energy and carbon sources for seed germination and biological metabolism^4,5^.

In plant cells, TAG synthesis occurs in three ways. The first is the assembly of free fatty acids and glycerol into TAG at the ER via the Kennedy pathway^1^, the second is the production of TAG and lysophosphatidylcholine (LPC) by transferring an acyl group from phosphatidylcholine (PC) to diacylglycerol (DAG)^6^, and the last is the reverse lipidotransferase (TA) transfer of one of the acyl groups from one diacylglycerol molecule to another diacylglcerol to form TAG and monoacylglycerol (MAG)^7^. In the classical Kennedy pathway, there are three major acyltransferases: GPAT (EC.2.3.1.15), 2-lysophosphatidic acid acyltransferase (LPAAT, EC.2.3.1.51) and diacylglycerol acyltransferase (DGAT, EC.2.3.1.20)^8–13^.

The enzyme activity of GPAT in plants was first observed in the mesocarp of avocado^14^. To date, three types of GPATs have been identified, which are located in the mitochondria, chloroplasts and endoplasmic reticulum (ER)^15–17^. In *Arabidopsis thaliana*, 10 genes have been shown to encode GPAT proteins, designated *GPAT1* to *GPAT9* and *ATS1*^18–20^. Glycerol-3-phosphate (G3P) is the carbon chain skeleton for the synthesis of TAG, and exhibits three sites for the attachment of fatty acids: sn-1, sn-2, sn-3^1,8^. GPAT9 is responsible for the first step of TAG synthesis, in which a fatty acid is transferred from acyl-CoA to the sn-1 site of glycerol-3-phosphate (G3P), forming lysophosphatidic acids (LPA)^8–13^. LPAAT transfers a second fatty acid to the sn-2-position of G3P to yield phosphatidic acid (PA) which is hydrolyzed by phosphatidic acid phosphatase to form diacylglycerol (DAG), after which the sn-3-position is acylated by DGAT to produce TAG^8^. Studies have shown that GPAT9 exhibits no phosphatase activity and that the majority of the acylation reactions catalyzed by this enzyme take place at the *sn*-1 position rather than the *sn*-2 position (5.3:1 ratio)^21^. GPAT9 is a membrane binding protein located in the ER; that exhibits *sn-*1 acyltransferase activity with a preference for acyl-CoA as its substrate, shares high homology with mGPAT3 and mGPAT4, which are related to animal fat synthesis, and it plays an important role in the synthesis of plant membrane lipids and storage lipids^21,22^. GPAT1 to GPAT8 are involved in the biosynthesis of cutin or suberin in plants; and are membrane-bound proteins^23–31^. ATS1 is a soluble protein located in chloroplasts, that uses acyl-ACP as a donor to catalyze the lipidoacylation of G3P and participates in lipid synthesis in the prokaryotic pathway^15,20^. There are four conserved acyltransferase motifs (I to IV) in GPAT proteins, which are critical for enzyme activity and G3P substrate binding. In motif I, His, Asp, Gly, and Pro are located at the catalytic center of the enzymes, and Phe, Arg and Glu in motif II and motif III are the binding sites of G3P^11,21^. In addition, GPAT1 to GPAT9 contain transmembrane domains (TMDs), that help GPATs anchor to the plasma membrane^17–31^.

In *Arabidopsis*, the overexpression of *AtGPAT9* results in an increase in seed weight, seed area and seed oil content, while the downregulation of *AtGPAT9* results in significant decreases in seed weight, seed area and seed oil content^21^. A knockout mutant of *AtGPAT9* shows pollen lethality and partial female gametophyte lethality, demonstrating that *GPAT9* contributes to lipid biosynthesis in developing pollen grains^6,9^. *LiGPAT* is found in the oleaginous green microalga *Lobosphaera incise*, and is a homologous gene of *AtGPAT9*; when *LiGPAT* was expressed heterogeneously in the model microalga *Chlamydomonas reinhardtii*, the TAG content was increased by 50%^32^. Similar results were found in a study of *JcGPAT2*, in which the overexpression of *JcGPAT2* in *Arabidopsis thaliana* led to a 43%-60% increase in oil content^33^. In summary, GPAT9 is responsible for the biosynthesis of TAG and plant membrane lipids in plants.

Peanut (*Arachis hypogaea* L.) is, an allotetraploid species (2n = 4x = 40, AABB)^34^ that is a major source of plant oil and protein, ranking as the fourth largest edible oilseed crop and the second most important grain legume in the world^35^. Oil content is an important quality trait for peanuts, and high oil content has always been a major goal in peanut breeding. When the seed oil content is increased by one percent, which is equivalent to a yield increase of 2%, the profit of the processing enterprise can be increased by 7%^36^. The oil content of different peanut cultivars ranges from approximately 41.86% to 56.37%^37,38^, which indicates great genetic potential for the breeding of new cultivars with both high oil content and yields. However, lipid synthesis in plants is a complicated biological process involving multiple genes, and exploring the key functional genes involved in peanut oil accumulation is important for both theoretical and practical applications. There have been some reports of the functional genes involved in TAG biosynthesis in peanut, including *GPAT9, LPAAT* and *DGAT*^39–42^. For example, the gene expression level of *AhLPAAT* in high-oil cultivar was found to be much higher than that in low-oil cultivar during seed development, and overexpression of *AhLPAT2* in *Arabidopsis* increased the seed oil content of the transgenic plants and the transcript levels of several key genes related to TAG assembly^40^. In a previous study, a *GPAT9* gene was isolated from peanut and shown to be highly expressed in stems, flowers and seeds; the maximum transcript accumulation of the *AhGPAT9* gene was observed at 50 DAP in the peanut cultivar Huayu19, and no *GPAT* genes involved in seed oil accumulation have been identified so far^39^.

*GPAT9* is responsible for TAG and plant membrane lipid biosynthesis. Peanut germplasm show a wide range of variation in seed oil content, but there has been no research assaying the allelic variation of *AhGPAT9* genes and its association with oil content in germplasm resources. In this study, a total of 171 peanut germplasm with different oil content were tested, and the main aims are: 1) to isolate and characterize *AhGPAT9* genes from peanut, 2) to analyze their expression patterns in different tissues and verify their functions in seed oil accumulation by gene transformation, and 3) to explore allelic polymorphisms in germplasm by sequencing and detect the elite haplotypes of *AhGPAT9* associated with high oil content.

## Results

### Isolation of *AhGPAT9* genes in peanut

**Figure 1:**
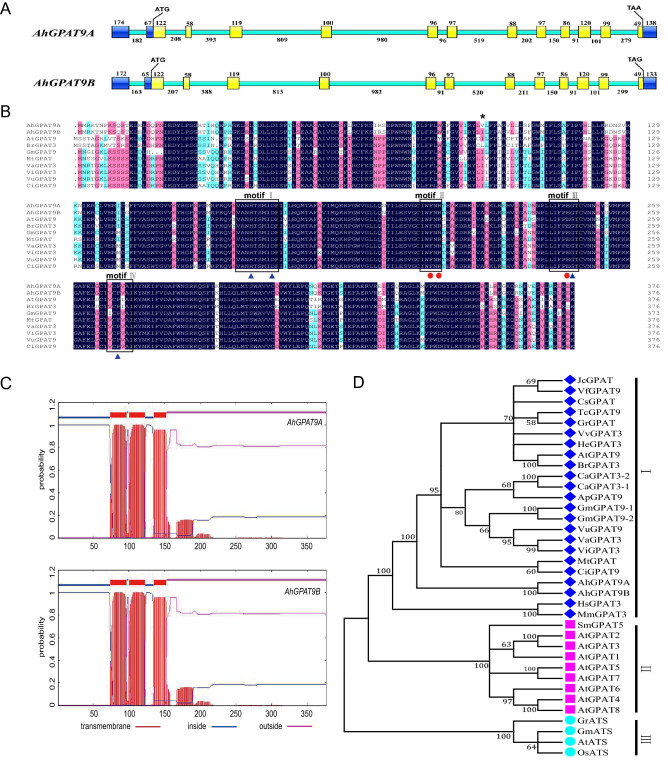
Sequence analysis of peanut *AhGPAT9* genes. (**A**) Gene structure of *AhGPAT9A* and *AhGPAT9B*. The UTRs are indicated with dark blue boxes, and the exons and introns are shown in yellow and green, respectively. The numbers are the sequence lengths. (**B**) Sequence alignment of AhGPAT9 proteins with related GPATs from other plants. "*" indicates an amino acid difference between AhGPAT9A and AhGPAT9B. The boxed motifs I, II, III and IV are conserved domains for acyltransferase. The catalytic activity centers are indicated by blue triangles, and the binding sites of the substrate are indicated by red circles. (**C**) Transmembrane domain prediction for AhGPAT9A and AhGPAT9B in TMHMM. The x-axis shows the amino acids, and the y-axis shows the posterior probabilities. (**D)** Phylogenetic tree based on the deduced amino acid sequences of GPATs. The amino acid sequences from other species are indicated as follows: At, *Arabidopsis thaliana*; Gm, *Glycine max*; Br, *Brassica napus*; Mt, *Medicago truncatula*; Vu, *Vigna unguiculata*; Vr, *Vigna radiate*; Va, *Vigna angularis*; Ci, *Cicer arietinum*; Gr, *Gossypium raimondii*; He, *Helianthus annuus*; Sm, *Selaginella moellendorffii*; Tc, *Theobroma cacao*; Vv, *Vitis vinifera*; Cs, *Citrus sinensis*; Cc, *Cajanus cajan*; Ap, *Abrus precatorius*; Vf, *Vernicia fordii*; Mm, *Mus musculus*; Jc, *Jatropha curcas*; Hs, *Homo sapiens*; Os, *Oryza sativa.*

Two *AhGPAT9* genes, designated *AhGPAT9A* and *AhGPAT9B*, were obtained from the A- and B- genomes of the peanut cultivar Fenghua2 (FH2), which were 5,537 bp and 5,516 bp in length, respectively, and the corresponding accession numbers in GenBank were MN124513 and MN124514. Both of these genes contained 13 exons and 12 introns, and they showed similar gene structures (Fig. 1A). The two genomic sequences shared a similarity of 95.65% with a total of 165 site differences, including substitutions, deletions and insertions (S1 Fig), 154 of which were present in intron regions. Among the 11 differences displayed in exon regions, nine were present in coding sequences, eight of which were synonymous mutations, while the other mutation resulted in a difference in the 95th amino acid (Fig. 1B).

The AhGPAT9A and AhGPAT9B, proteins encoded by two *AhGPAT9* genes, both contained 376 amino acids. Their molecular weight (MW) was 43.5 kDa. The isoelectric point (pI) was 9.09, and the grand average of hydropathicity (GRAVY) was -0.110. Secondary structure prediction showed that AhGPAT9A consisted of 46.01% α helices, 15.96% extended strands, 3.72% β turns and 34.31% random coils, while AhGPAT9B consisted of 43.35% α helices, 17.82% extended strands, 3.46% β turns and 35.37% random coils. Four conserved acyltransferase domains were identified in AhGPAT9, which are important for acyltransferase activity (Fig. 1B). These domains were designated motif I (VANHTSMIDF), motif II (IWFNR), motif III (IFPEGT) and motif IV (VCPVAI). The catalytic activity centers were His171 and Asp176 in motif I, Gly246 in motif III, and Pro270 in motif IV. The binding sites of the substrate glycerol-3-phosphate (G3P) were Phe213 and Arg215 in motif II, and Glu245 in motif III. It was predicted that the N-terminus of AhGPAT9 contained three TMDs (Fig. 1C) using TMHMM software.

To reveal the relationships of the *GPAT* genes, a phylogenetic tree was constructed using GPAT proteins from different plants (Fig. 1D). All GPATs were divided into three main clades, and AhGPAT9 together with GPAT9 proteins from other plants were grouped in clade I, which consisted of ER-localized proteins, including AtGPAT9 (*Arabidopsis thaliana*), GmGPAT9 (*Glycine max*), VuGPAT9 (*Vigna unguiculata*), etc. The GPAT1 to GPAT8 proteins were clustered in clade II, which comprised membrane-bound proteins; while the ATS proteins were grouped in clade III, which comprised soluble proteins located in the chloroplast.

Overall, there were few differences in the primary and advanced structures of the two AhGPAT9 proteins, and they may have similar functions.

### Overexpression and antisense transformation of *AhGPAT9* genes

**Figure 2:**
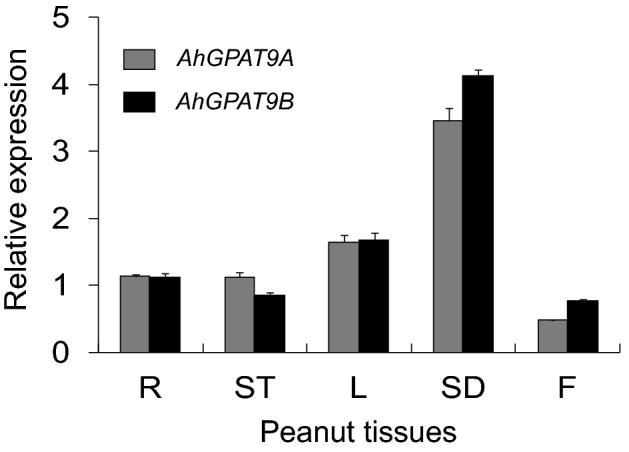
Expression analysis of *AhGPAT9* using qRT-PCR in different peanut tissues and at seed development stages. R, root; ST, stem; F, flower; SD, seed. The relative mRNA abundance was normalized with respect to that of peanut *AhACT11*. The bars indicate the standard deviations (*SD*) of three replications.

The analysis of the relative expression of *AhGPAT9* genes by quantitative real-time PCR (qRT-PCR) using *AhACT11* as a reference revealed that the two genes exhibited specific temporal and spatial expression patterns in different tissues and that the seeds exhibited the highest transcript accumulation (Fig. 2). The expressions of the *AhGPAT9* genes reached the maximum value at 42 DAP, which was consistent with the oil accumulation rate in peanut seeds. These results suggested that *AhGPAT9* may play important roles in peanut seeds.

**Figure 3:**
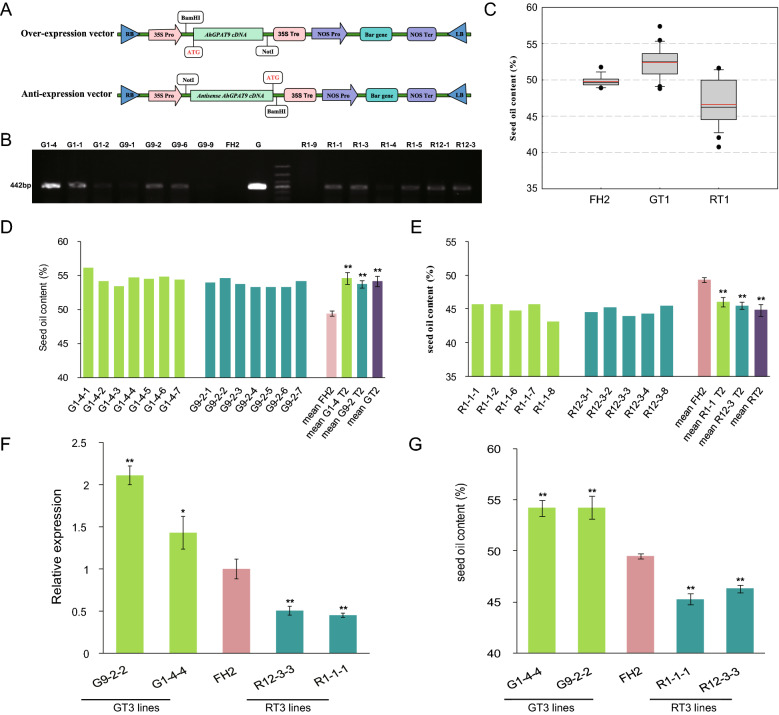
Identification of putative *AhGPAT9* transgenic plants and the seed oil contents of transgenic plants. (**A**) The expression vectors containing the sense and anti-sense sequences of the *AhGPAT9* gene. Sense and anti-sense *AhGPAT9* gene fragments were inserted into the pGPVE vector between the *BamH*I and *Not*I restriction sites. LB, left border; RB, right border; NOS Pro is the promoter of the nopaline synthase gene; *Bar* gene, phosphinothricin acetyltransferase; 35S, cauliflower mosaic virus 35S promoter. (**B**) Identification of transgenic plants showing overexpression and antisense-expression of the *AhGPAT9* genes. G represents the recombinant plasmids, M is a 1,000 bp DNA marker and FH2 represents the wild-type (WT). G1-4, G1-1, G1-2, G9-2, G9-6, G9-1, and G9-6 were OE transgenic plants, and R1-9, R1-1, R1-3, R1-4, R1-5, R12-1 and R12-3 were AE transgenic plants. (**C**) Oil content distribution of transgenic T_1_ seeds. FH2, wild-type control including 14 plants; GT1, *AhGPAT9-*overexpressing transgenic T_1_ peanut line including 20 plants; RT1, antisense-expressing transgenic T_1_ peanut line including 24 plants. The box contains 50% of the data points. The black and red bars across the boxes represent the medians and means, respectively. The top and bottom ends of the ‘whiskers’ represented the highest and lowest values observed, respectively. Black dots represent outliers. (**D**) Seed oil content of T_2_ *AhGPAT9* overexpressing transgenic peanut plants. Mean G1-4 T2 indicates the mean of seven G1-4 transformants; Mean G9-2 T2 indicates the mean of seven G9-2 transformants; Mean G T2 indicates the mean of 14 G1-4 and G9-2 transformants. Values are the average seed oil percentage ± SD (*n* = 7 and 7 for G1-4 and G9-2, respectively). (**E**) Seed oil content of homozygous T_2_ *AhGPAT9* anti-sense expressing transgenic peanut plants. Mean R1-1 T2 indicates the mean of five R1-1 transformants; Mean R12-3 T2 indicates the mean of seven R12-3 transformants; Mean R T2 indicates the mean of 10 R1-1 and R12-3 transformants. Values are the average seed oil percentage ± SD (*n* = 5 and 5 for R1-1 and R12-3, respectively). (**F**) Expression analysis of *AhGPAT9* in T_3_ transgenic lines. Total RNA was prepared from the transgenic lines. Gene expression levels are shown relative to the expression of *AhACT11* in each sample. Values are means ± SDs (n = 3). The transcription level of each gene in the wild-type FH2 was set as 1. Asterisks indicate significant differences between the FH2 and transgenic lines at p < 0.01 (**) and p < 0.05 (*). (**G**) Effect of *AhGPAT9* overexpression and antisense expression on the seed oil content of T_3_ lines. Values are means ± SDs (n = 10).

To clarify the gene function of *AhGPAT9* in oil accumulation process of peanut seeds, we constructed an overexpression vector (AhGPAT9-OE) and an anti-sense expression vector (AhGPAT9-AE) (Fig. 3A) and introduced the two constructs into *Agrobacterium tumefaciens*, which was subsequently used to transform the FH2 cultivar. The presence of the *AhGPAT9* transgene was identified in the T_0_, T_1_ and T_2_ generations by PCR (Fig. 3B). In 2016, a total of 20 AhGPAT9-OE T_1_ (GT1) plants and 24 AhGPAT9-AE T_1_ (RT1) plants were generated. Data analysis showed that the seed oil content of 20 GT1 transgenic peanuts ranged from 48.79% to 57.38%, with a mean value of 52.42%. Compared with the oil content of wild-type FH2, which exhibited an oil content of 49.76% on average, the oil content was increased by 5.35% in GT1 plants. There were 16 GT1 plants with a higher seed oil content than the wild-type (Fig. 3C). In contrast, the seed oil content of 24 RT1 plants ranged from 40.74% to 51.62%, with a mean value of 46.43%, representing a decrease of 6.70% compared to the wild-type plants. There were 18 RT1 plants showing a lower seed oil content than FH2 (Fig. 3C).

In 2017, the seed oil contents of the GT2 and RT2 transgenic plants were measured. The average oil content of seven GT2 plants derived from G1-4 was 54.58%, and it was 53.73% for seven GT2 plants derived from G9-2. The observed contents were all significantly higher than that of the wild-type (Fig. 3D). The mean oil content of the 14 GT2 transgenic plants was 54.16%, representing an increase of 8.82% compared with wild-type FH2 (Fig. 3D). In addition, the average oil content of five RT2 plants derived from R1-1 was 45.01%, and it was 44.73% to other five RT2 plants derived from R12-3, which all showed significantly lower oil contents than the wild-type plants (Fig. 3E). The mean oil content of ten RT2 plants was 44.87%, corresponding to a decrease of 9.13% than the wild-type plants (Fig. 3E).

Two GT2 plants (G1-4–4 and G9-2–2) and two RT2 plants (R1-1–1 and R12-3–3) were selected to produce the GT3 and RT3 transgenic lines respectively, in 2018. We tested the expression of the *AhGPAT9* gene in transformed peanut plants (G1-4–4, G9-2–2, R1-1–1 and R12-3–3) using qRT-PCR with gene-specific primers. As shown in Fig. 3F, *AhGPAT9* expressions in the overexpression lines (G1-4–4 and G9-2–2) were much higher than that in wild-type FH2. In contrast, the expression levels of the *AhGPAT9* gene in anti-sense-expressing peanut transgenic lines (R1-1–1 and R12-3–3) were lower than those in FH2 (Fig. 3F). In general, the expression levels of the *AhGPAT9* gene in the overexpression plant lines (G1-4–4 and G9-2–2) were higher than those lines R1-1–1 and R12-3–3 (Fig. 3F). We also measured the total lipid contents of FH2 seeds and seeds from the *AhGPAT9*-overexpressing lines and *AhGPAT9*-anti-sense expressing lines. The oil content of the G1-4–4 lines was 54.21%, and it was 54.27% for the G9-2–2 lines, representing increases of 9.55% and 9.67% compared to wild-type FH2, respectively (Fig. 3G). The mean oil content of the two GT3 lines was 54.24%, which was 4.75% higher than that of the wild-type (49.49%, Fig. 3G). In contrast, the oil content of the R1-1–1 lines was 45.29%, and it was 46.31% for the R12-3–3 lines, representing decreases of 8.48% and 6.42% compared to wild-type FH2, respectively; the mean oil content of the two RT3 lines was 45.80%, which was significantly lower than that of wild-type FH2 (Fig. 3G). Some phenotypic traits of the GT3 and RT3 transgenic lines were also measured. Compared with wild-type FH2, there were no significant differences in the main stem height, lateral branch length, pod length or seed size in the transgenic lines (S2 Fig). These results indicated that overexpression or antisense expression of *AhGPAT9* had little influence on plant growth, but seed oil accumulation could be promoted by the overexpression and suppressed by the antisense inhibition of *AhGPAT9*.

### Allelic polymorphism analysis of *AhGPAT9* in peanut germplasm

Peanut germplasm resources exhibit great differences in oil content. The sites of sequence polymorphism in *AhGPAT9A* identified in 171 peanut germplasm are summarized in S1 Table, and the allelic polymorphism information of *AhGPAT9B* is supplied in S2 Table.

**Figure 4:**
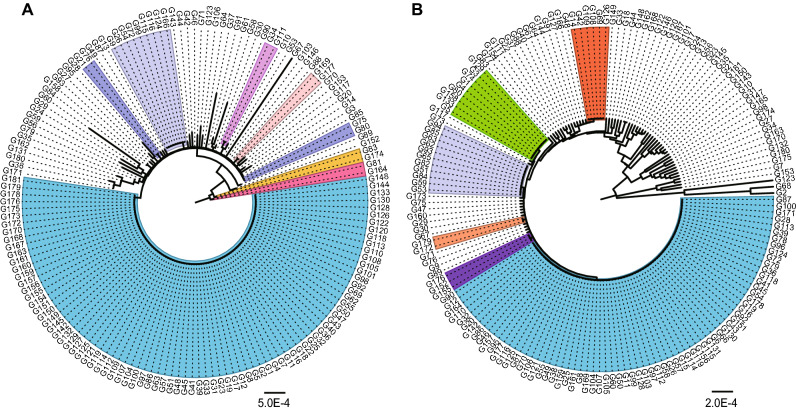
Clustering analysis of 171 peanut germplasm based on allelic polymorphisms from *AhGPAT9A* (**A**) and *AhGPAT9B* (**B**). The germplasm of the same haplotype are indicated in one color, and the *a1* and *b1* haplotypes are colored in blue.

A total of 118 polymorphic sites from *AhGPAT9A* were identified, including 92 SNP sites and 26 InDels, with one SNP occurring every 60 bp and one InDel every 213 bp on average, and thirteen SNPs in exon regions caused amino acid changes (S1 Table). The frequency of polymorphic sites in *AhGPAT9A* in the 171 peanut germplasm ranged from 0.58% to 12.28%. The nucleotide diversity (i.e., the average pairwise sequence differences between two random sequences in one sample^43^) in the sequenced region of *AhGPAT9A* measured by π (pairwise nucleotide diversity) was 0.00085, and the variable frequency at per site (Theta) in *AhGPAT9A* was 0.00364. Tajima’s *D* statistic was estimated to test whether the SNPs were neutral mutations^43,44^, and the *D* value for *AhGPAT9A* was -2.41743, which was extremely significant (*p* < 0.01), indicating that the nucleotide variations in *AhGPAT9A* could not have been a result of the standard neutral selection. Allelic polymorphism analysis of *AhGPAT9A* showed that the 171 peanut germplasm could be divided into 64 types, designated haplotypes *a1* to *a64*, among which 86 materials were of the *a1* type (Fig. 4A). In *AhGPAT9B*, a total of 94 polymorphic sites were identified, including 72 SNPs and 22 InDels (S2 Table); on average, one SNP could be detected every 77 bp, and one InDel could be detected every 251 bp. The frequency of polymorphic sites in *AhGPAT9B* ranged from 0.58% to 13.45%. The nucleotide diversity π was 0.00129 among the 171 peanut germplasm, and the value of Theta was 0.00316. The Tajima’s *D* value for *AhGPAT9B* was -1.87261, which was also statistically significant (*p* < 0.05). Allelic polymorphism analysis of *AhGPAT9B* showed that the 171 peanut germplasm could be divided into 75 haplotypes, designated *b1* to *b75*, among which 71 materials were of the *b1* type (Fig. 4B).

### *AhGPAT9*-encoded amino acid sequence analysis in peanut germplasm

**Figure 5:**
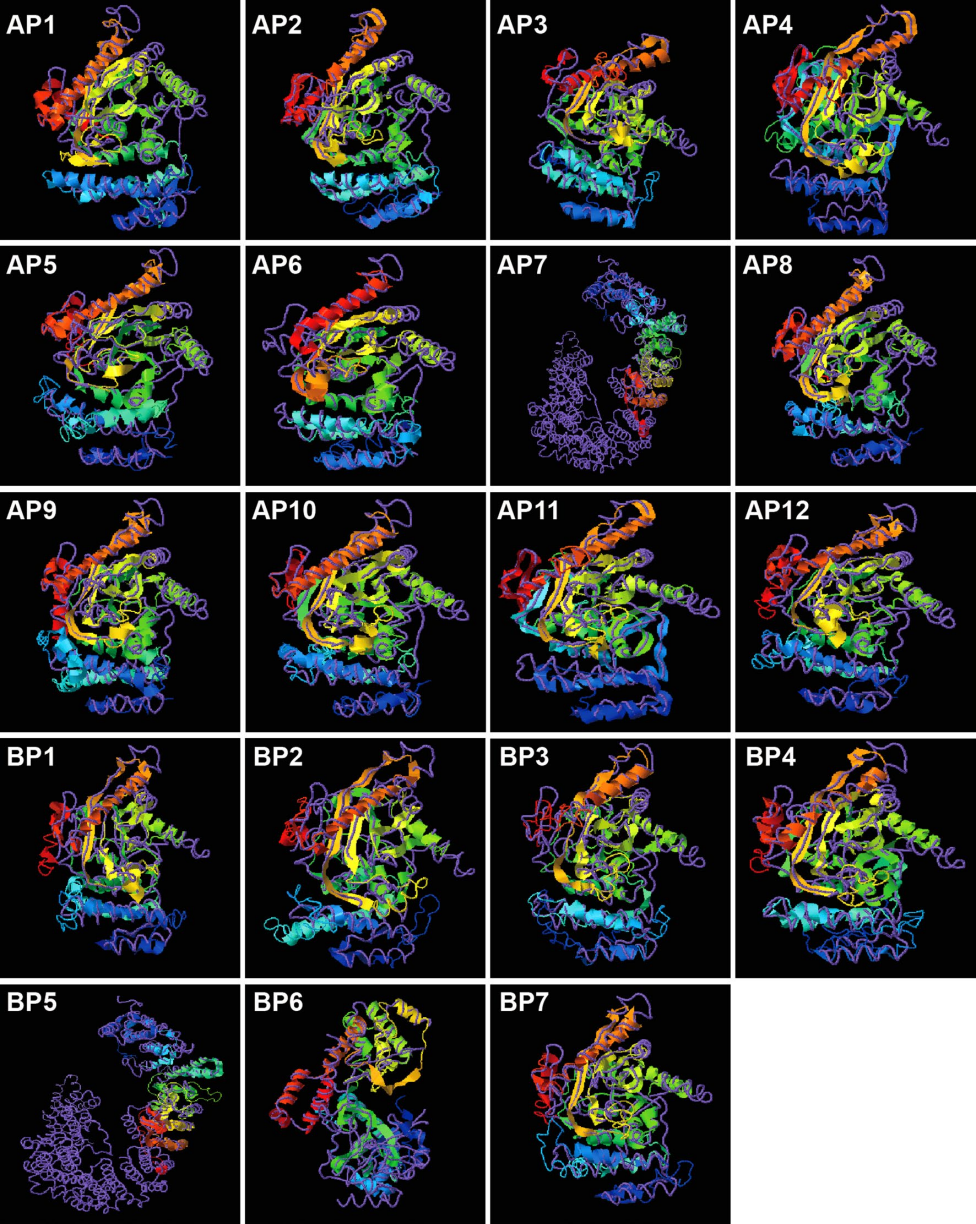
Proteins spatial structure prediction of AhGPAT9. AP1 to AP12 were obtained from *AhGPAT9A*, and BP1 to BP7 were obtained from *AhGPAT9B*. The spatial structures were predicted by using the online software I-TASSER (https://zhanglab.ccmb.med.umich.edu/I-TASSER/).

Many of the polymorphisms of *AhGPAT9* were identified in intron regions, and many of the mutations in coding regions were synonymous. The 64 *AhGPAT9A* haplotypes produced 12 protein types, designated AP1 to AP12. Sequence analysis showed that the amino acid sequences encoded by *AhGPAT9A* in 157 peanut accessions were identical to AP1, which used as the wild-type. Thirteen non-synonymous substitutions detected in the exon region of *AhGPAT9A* caused amino acid differences in 14 peanut accessions, and those changes resulted in 11 mutant proteins (Table 1). AP10 contained three mutated amino acids, p.E13G, p.L104S and p.K130R. AP4 contained two mutated amino acids, p.R162Q and p.T184P, resulting from Ag.2874G > A and Ag.3035A > C substitutions, respectively, which were located in the acyltransferase domain. AP2, AP3, AP5, AP6, AP8, AP11 and AP12 contained only one mutated amino acid (Table 1). The Ag.4595G > T substitution in AP6 caused coding amino acid no.346 to become a stop codon, and there were 31 fewer amino acids in the product than in AP1. Protein spatial structure prediction was performed by using the online software I-TASSER^45–47^. As shown in Fig. 5, compared with AP1, the spatial structures of most mutant proteins showed little difference, while AP4, AP6, AP7 and AP11 were significantly different from AP1. Detailed information showed that AP7 lacked both the binding sites for the G3P substrate and the enzyme catalytic centers, while AP11 had five more G3P binding sites (Table 1). Although their spatial structures showed little difference, AP2 lacked binding sites for substrate G3P, while AP3 had two fewer binding sites, including the typical Arg330 site, whereas AP12 had three more G3P binding sites (Table 1).

**Table 1:** Characteristics of different protein types encoded by *AhGPAT9* in peanut germplasm.

Protein type	Non-synonymous mutations	Amino acid differences	G3P binding sites	Catalytic centers
AP1	–	–	Met256, **Phe257**, Lys259, Ile323, Ala326, **Glu327**, **Arg330**	His171, Asp176
AP2	Ag.4941 T > C	p.F365S	–	His171, Asp176
AP3	Ag.1789G > A	p.S127N	**Phe115**, Ile116, His119, Leu121, **Glu206**	His171, Asp176
AP4	Ag.2874G > A Ag.3035A > C	p.R162Q· p.T184P·	Gly56, Ala57, Asp60, Ser62, **Glu206**, Ala261, **Phe262**, **Glu327**, **Arg330**, **Glu331**, Ser334	His171, Asp176
AP5	Ag.44A > G	p.E15G	Met256, **Phe257**, Lys259, Ile323, Ala326, **Glu327**, **Arg330**	His171, Asp176
AP6	Ag.4595G > T	p.G346*	Met256, **Phe257**, Lys259, Ile323, Ala326, **Glu327**, **Arg330**	His171, Asp176
AP7	Ag.4947A > G Ag.4950C > T	p.E367G p.S368F	–	–
AP8	Ag.38A > G	p.E13G	Met256, **Phe257**, Lys259, Ile323, Ala326, **Glu327**, **Arg330**	His171, Asp176
AP9	Ag.1720 T > C Ag.1798A > G	p.L104S p.K130R	Met256, **Phe257**, Lys259, Ile323, Ala326, **Glu327**, **Arg330**	His171, Asp176
AP10	Ag.38A > G Ag.1720 T > C Ag.1798A > G	p.E13G p.L104S p.K130R	Met256, **Phe257**, Lys259, Ile323, Ala326, **Glu327**, **Arg330**	His171, Asp176
AP11	Ag.2804A > G	p.M139V	Gly56, Ala57, Asp60, Ser62, **Glu206**, Met256, **Phe257**, Lys259, **Glu327**, **Arg330**, **Glu331**, Ser334	His171, Asp176
AP12	Ag.883G > A	p.V95I	Val118, His119, Leu122, Ile204, Met256, **Phe257**, Ile323, Ala326, **Glu327**, **Arg330**	His171, Asp176
BP1	–	–	Val118, His119, Leu122, Ile204, Met256, **Phe257**, Ile323, Ala326, **Glu327**, **Arg330**	His171, Asp176
BP2	Bg.4328 T > C Bg.4344 T > C	p.L291F p.L296P	Met256, **Phe257**, Lys259, Ile323, Ala326, **Glu327**, **Arg330**	His171, Asp176
BP3	Bg.4323A > G	p.Q289R	Met256, **Phe257**, Lys259, **Glu327**, **Arg330**, **Glu331**, Ser334	His171, Asp176
BP4	Bg.4344 T > C	p.L296P	Met256, **Phe257**, Lys259, Ile323, Ala326, **Glu327**, **Arg330**	His171, Asp176
BP5	Bg.4193A > G	p.N276S	–	–
BP6	Bg.1747G > A	p.A114T	–	–
BP7	Bg.1733 T > C	p.M109T	–	His171, Asp176

Protein types AP1 to AP12 were obtained from *AhGPAT9A*, while BP1 to BP7 were obtained from *AhGPAT9B*, and their amino acid differences compared with AP1 and BP1, respectively, were determined. p.F365S indicates a change from phenylalanine to serine at amino acid no.365 in AP2. · indicates that the amino acid changes were located in the acyltransferase domain. * indicates that the amino acid was terminal. The amino acids **Phe**, **Glu** and **Arg** in bold format represent typical G3P binding sites in GPAT9 proteins.

The 75 *AhGPAT9B* haplotypes produced seven protein types, designated BP1 to BP7. Sequence analysis showed that the amino acid sequences encoded by *AhGPAT9B* in 165 peanut accessions were identical to BP1, which was used as the wild-type. Six non-synonymous substitutions detected in exon regions caused amino acid differences in six peanut accessions, and those changes resulted in six mutant proteins (Table 1). BP2 contained two mutated amino acids, p.L291F and p.L296P, resulting from Bg.4328 T > C and Bg.4344 T > C substitutions, respectively, and these changes led to great differences in the protein spatial structures (Fig. 5). Due to the presence of one mutated amino acid in BP5 and BP6, their protein spatial structures were significantly different from that of BP1, and they lacked both the binding sites for substrates the G3P substrate and the enzyme catalytic centers (Table 1, Fig. 5). Although BP7 lacked G3P binding sites because of the mutation of the -p.M109T amino acid, its spatial structure showed little difference from BP1 (Table 1, Fig. 5).

### Detection of oil content in peanut germplasm

**Figure 6:**
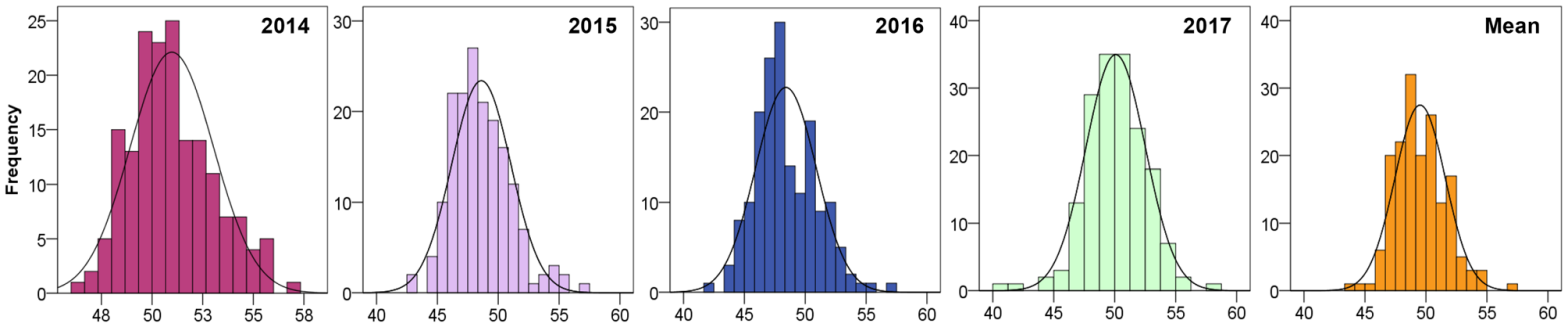
Phenotypic distribution histograms of seed oil content in peanut germplasm. The x-axis shows groups with different oil content ranges, and the y-axis shows the number of lines in each group.

The oil content of 171 peanut germplasm was tested from 2014 to 2017 (Table 2), among which the highest oil content was 57.78%, while the lowest oil content was 40.01%, showing a wide range among peanut germplasm. The broad-sense heritability (*h*_*B*_^2^) of the oil content was 84.6%, and the coefficient of variation (CV) values were all around 5%, showing that the oil content was mainly determined by the genotype and was less influenced by the environment. The average oil content of the 171 peanut varieties was 49.52%, and the phenotypic distribution histograms across four consecutive years showed near normality (Fig. 6), indicating that there were some major genes controlling peanut oil traits and that *AhGPAT9* may be one of the most important genes.

**Table 2:** Detection of oil content in 171 peanut germplasm.

Year	Min	Max	Mean	*SD*	*CV* (%)	*Sk*	*Ku*	*h*_*B*_^2^ (%)
2014	46.52	56.89	50.97	2.06	4.03	0.43	− 0.17	84.6
2015	42.41	57.29	48.41	2.50	5.16	0.51	0.30	
2016	42.65	56.93	48.61	2.43	5.00	0.62	0.74	
2017	40.01	57.78	50.08	2.44	4.87	− 0.34	1.71	
Mean	43.73	56.89	49.52	2.07	4.18	0.40	0.56	

*SD*, standard deviations; *CV*, coefficient of variation; *Sk*, skewness; *Ku*, kurtosis; *h*_*B*_^2^ = [V_G_/(V_G_ + V_E_)] × 100%, where V_G_ is genotypic variance and V_E_ is environmental variance.

### Analysis of different *AhGPAT9* haplotypes associated with oil content

The oil content of 86 *a1* haplotype peanut germplasm varied from 43.73% to 56.89%, with an average oil content of 49.67%. The oil content of 71 *b1* germplasm varied from 45.72% to 54.72% with an average of 49.48%. Peanut is an allotetraploid species comprising both A- and B- genomes; thus, the site effects of *AhGPAT9A* and *AhGPAT9B* were difficult to calculate separately. Based on the polymorphism analysis of both *AhGPAT9A* and *AhGPAT9B*, all the germplasm could be divided into 109 types, among which 39 were of the *a1b1* combination type. The oil content of *a1b1* germplasm varied from 46.62% to 54.72% with an average oil content of 49.85%.

In the germplasm with the combined *a*- and *b1* (*a*-/*b1*) haplotype, *a5* showed a positive phenotypic effect of 9.23% (Table 3), and the oil content was 54.05%, while the oil content of the *a7*, *a14* and *a48* germplasm were significantly lower than the mean value of *a*-/*b1*, with negative phenotypic effects of 3.32%, 5.79% and 7.61% (Table 3), respectively. However, among the *a1*/*b*- germplasm, the oil content of the *b57*, *b30* and *b35* germplasm were 56.89%, 54.59% and 53.98%, respectively, which were significantly higher than the average of *a1*/*b*- germplasm, with positive phenotypic effects of 14.54%, 9.91% and 8.68% (Table 3), respectively, while the *b51* and *b54* germplasm exhibited negative effects of 10.83% and 11.95% with oil content of 44.29% and 43.73% (Table 3), respectively.

**Table 3:** Elite haplotype selection of *AhGPAT9* for oil content in peanut germplasm.

Type	Haplotype combination	Protein combination	Oil content (%)	Phenotypic effect (%)
*a* haplotype	*a5/b1*	AP1/BP1	54.05 ± 0.97aA	9.23
	*a7/b1*	AP2/BP1	47.84 ± 2.04cBC	− 3.32
	*a14/b1*	AP12/BP1	46.62 ± 1.99cdCD	− 5.79
	*a48/b1*	AP1/BP1	45.72 ± 1.30dD	− 7.61
	*a-/b1*	–	49.48 ± 1.12bB	–
*b* haplotype	*a1/b57*	AP1/BP1	56.89 ± 0.81aA	14.54
	*a1/b30*	AP1/BP1	54.59 ± 0.70bAB	9.91
	*a1/b35*	AP1/BP1	53.98 ± 1.19bB	8.68
	*a1/b5*	AP1/BP7	49.71 ± 1.75cC	0.09
	*a1/b51*	AP1/BP1	44.29 ± 1.31dD	− 10.83
	*a1/b54*	AP1/BP1	43.73 ± 1.71dD	− 11.95
	*a1/b−*	–	49.67 ± 1.03cC	–
*a*/*b* combination	*a62/b10*	AP1/BP1	53.60 ± 1.67aA	7.52
	*a38/b31*	AP3/BP6	52.70 ± 1.25bAB	5.72
	*a43/b36*	AP4/BP1	52.34 ± 1.47bB	5.00
	*a9/b42*	AP7/BP1	45.93 ± 1.31dD	− 7.86
	*a1/b1*	–	49.85 ± 1.11cC	–

The phenotypic effects suggested that the oil content of germplasm of a certain type (*a*−/*b*−) was increased or decreased compared with the references in the same groups, which were *a*−/*b*1 for type *a*, *a1*/*b*– for type *b*, and *a1*/*b1* for type *a*/*b*. Different capital and lowercase letters indicate significant differences within groups at *p* < 0.01 and *p* < 0.05, respectively.

In addition, the oil content of the germplasm with haplotype combinations *a62*/*b10*, *a38*/*b31* and *a43*/*b36* were significantly higher than those of *a1*/*b1* germplasm with a positive effect value of more than 5%, while the oil content of the *a9*/*b42* germplasm was 45.93% (Table 3), which was significantly lower than that of *a1*/*b1* germplasm. Therefore, for the *AhGPAT9* genes, we speculated that *a5*, *b57*, *b30* and *b35* were elite haplotypes associated with high oil content, while *a7*, *a14*, *a48*, *b51* and *b54* were low oil haplotypes. Additionally, *a62*/*b10*, *a38*/*b31* and *a43*/*b36* were haplotype combinations associated with high oil content, but *a9*/*b42* was a low oil combination.

## Discussion

GPATs are important enzymes involved in different metabolic pathways in plants and their conserved sequences contain four acyltransferase motifs (PF01553), which are critical for catalyzing and binding to G3P substrates. It has been suggested that motifs II and III are important for substrate binding, while motifs I and IV are responsible for catalysis^48^. GPAT9 is a large glycerolipid acyltransferase family^11^. In *Arabidopsis thaliana*, the *AtGPAT9* gene plays an essential role in the synthesis of storage lipids and membrane lipids^18,19,21,29^. In our study, peanut *AhGPAT9* genes were obtained, which were homologues to the *AtGPAT9* gene; the amino acid sequences of the AhGPAT9 proteins showed high sequence similarity to AtGPAT9, and they displayed much closer evolutionary relationships with mGPAT3 and mGPAT4 of mammals (Fig. 1D), which have been confirmed to play distinct roles in adipogenesis^26^. The results suggested that *AhGPAT9* genes may exhibit similar functions in the synthesis of storage lipids in peanut.

The gene expression patterns across different peanut tissues showed that transcript accumulation was highest in seeds, and that the expressions of the *AhGPAT9* genes reached the maximum value at 42 DAP, consistent with Chi’s research^39^. These results showed that the expression of *AhGPAT9* gene was in accord with the lipid accumulation rate in peanut seeds, indicating potential roles of *AhGPAT9* in seed development (Fig. 2). Furthermore, the allele type *a1* was used to conduct gene transformation, compared with wild-type FH2, the seed oil content of AhGPAT9-OE transgenic plants was significantly increased. In *a* haplotype accessions, the allele contributing for the highest oil content was *a5*, and the oil content was 54.05% (Table 3), while that of the over-expressing lines was 54.24% (Fig. 3G). So, the effect of gene over-expression can make the common oil content germplasm increase to a higher oil level, and the oil content of over-expressing lines can reach the highest oil content with that of the best allele. While it was significantly decreased in the AhGPAT9-AE transgenic peanut lines, which oil content was 45.80% (Fig. 3G). The allele contributing for the lowest oil content was *a48* (Table 3), the oil content of the accessions carrying *a48* was 45.72% (Table 3), while that of the anti-sense expressing lines was 45.80% (Fig. 3G). So, the effect of gene down-regulated expression can make the common oil content germplasm decrease to a lower oil level, and the oil content of anti-sense expressing lines was almost equal to that of the germplasm with the allele contributing for the lowest oil. The results indicated that the *AhGPAT9* genes that we obtained indeed play a key role in the accumulation of seed oil in peanut. In addition, as an important part of our transgenic work, research on gene copy number and integration site of AhGPAT9 genes is still in progress now, and we hope that the gene effects on target traits can be further clarified. Recent studies have shown that the seed oil content can be decreased by 26% to 44% in *Arabidopsis* through the *AtGPAT9* knockout method^19^, and *AtGPAT9* down-regulation also causes significant decreases in oil content in GPAT9-RNAi lines^21^. Compared with the wild-type, the overexpression of *AtGPAT9* in *Arabidopsis* not only increased the seed oil content significantly, but also increased the TAG content in the leaves of the transgenic lines by 153.3%^21^.

However, oil content is a quantitative trait with a complex underlying genetic mechanism. Lipid synthesis in plants is a complicated biological process involving multiple genes. In *Arabidopsis*, more than 120 enzymatic reactions and 600 genes are involved in oil accumulation^1^, which is regulated by TAG synthesis pathways, carbon metabolism, FA synthesis, and even cell differentiation^1,34^. In our study, 171 peanut germplasm were tested for oil content across four consecutive years, and their oil content varied from 40.01% to 57.78%, showing a wide range (Fig. 6). The high broad-sense heritability (*h*_*B*_^2^) and low coefficient of variation (*CV*) values obtained suggested that oil content is mainly determined by genotype, which is consistent with previous studies^38,49^. Although the results from different researchers regarding the genetic dissection of the oil content trait differ considerably, there must be major genes controlling oil content, and *GPAT9* may be one of the most important genes. The allelic polymorphism analysis of *AhGPAT9* in peanut germplasm will provide us with elite alleles and valuable information for breeding new lines with higher oil content.

Peanut is an allotetraploid species (AABB); thus, two *GPAT9* genes were identified among 171 peanut cultivars in our study: *AhGPAT9A* from the A-genome and *AhGPAT9B* from the B-genome. A total of 118 allelic polymorphic sites from *AhGPAT9A* were identified, together with 94 variation sites from *AhGPAT9B*, including SNPs and InDels (S1 Table and S2 Table). The Tajima’s *D* value for the SNPs in *AhGPAT9A* was -2.41743, whereas it was -1.87261 for *AhGPAT9B*, which were both statistically significant (*p* < 0.05), indicating that the nucleotide variations in the *AhGPAT9* genes were not caused by standard neutral selection and that *AhGPAT9B* may be subject to greater artificial selection pressure. Based on sequence polymorphic analysis, the *AhGPAT9A* genes of the 171 peanut germplasm showed 64 haplotypes (*a1* to *a64*), while 75 haplotypes were identified in *AhGPAT9B* (*b1* to *b75*). In our study, 86 of 171 peanut germplasm were of the *a1* type, while 71 were of the *b1* type; thus, haplotypes *a1* and *b1* were hypothesized to be wild-type haplotypes of the *AhGPAT9A* and *AhGPAT9B* genes, respectively (Fig. 4). For these two genes, the 171 peanut varieties could be divided into 109 combination types, among which 39 varieties were of the *a1b1* types. The analysis of the deduced amino acid sequence of *AhGPAT9* showed that 64 *AhGPAT9A* haplotypes produced 12 protein types (AP1 to AP12), while 75 *AhGPAT9B* haplotypes produced seven protein types (BP1 to BP7). Many allelic polymorphisms were located in the intron region, and many mutations in the coding regions were synonymous; thus, sequence analysis showed that the amino acid sequences encoded by *AhGPAT9A* in 157 peanut accessions were identical to AP1, including 52 *a*- haplotypes (S1 Table). There were 14 peanut accessions that showed a total of 11 mutant proteins (Table 1). Regarding the proteins encoded by *AhGPAT9B*, sequence analysis showed that 165 peanut accessions were identified as BP1-type accessions, including 69 *b*- haplotypes (S2 Table), and the remaining varieties showed six mutant proteins (Table 1).

To study the relationship between the *AhGPAT9* haplotypes and oil content, statistical and differential analyses were performed to explore elite high-oil or low-oil haplotypes or haplotype combinations. As a result, *a5*, *b57*, *b30* and *b35* showed significantly positive phenotypic effects, and they were considered elite high-oil haplotypes, while *a7*, *a14*, *a48*, *b51* and *b54* were speculated to be low-oil haplotypes. Furthermore, *a62*/*b10*, *a38*/*b31* and *a43*/*b36* were the best haplotype combinations for high oil content, but *a9*/*b42* was a low-oil combination (Table 3). The deduced protein sequence analysis also suggested some correlations. For example, the Ag.4941 T > C mutation in *a7* caused a p.F365S amino acid change in AP2, which lacked binding sites for the G3Psubstrate, and enzyme activity might have been decreased to some level (Table 1). The Ag.2874G > A and Ag.3035A > C mutations in *a43* caused amino acid changes in the acyltransferase domain of the AP4 protein, for which the spatial structure was also significantly different, and the mutation resulted in the presence of more G3P binding sites in AP4 (Table 1). However, the oil content of the different haplotypes varied significantly and showed great differences even for the same haplotype. For example, the oil content of the *a1* varieties varied from 43.72% to 56.89%, while it varied from 45.72% to 54.72% for the *b1* varieties, but the mean values were both around 49%, which was the average level for the entire population (S3 Table). The reason may be that oil accumulation is a complex process involving many reactions and regulatory steps^1^. In addition, cultivated peanut is an allotetraploid (AABB) species comprising two genomes with a high repetitive DNA content and similar genes may have redundant functions^50,51^. A previous study suggested that the homologous *ahFAD2A* and *ahFAD2B* genes showed significant additive effects and exhibited multiple effect interactions for regulating the contents of palmitic acid, oleic acid, and linoleic acid, and the O/L ratio^52,53^. In *Brassica napus*, there are three functional *FAD2* genes and on non-functional *FAD2* gene^54^. Among the three homologous genes of wheat, *TaGW2-6B* has a greater effect on the one-thousand kernel weight (TKW) than *TaGW2-6A*; an additive effect has been identified between them, and the combination *6A-A*/*6B-1* is the most effective^55^. Therefore, in the process of TAG biosynthesis, two *AhGPAT9* genes may exert an additive effect, or complementary effects, or only one of them may play a role. *AhGPAT9* affects oil content in peanut, but it is not the sole gene determining this trait. Similar results were found in a polymorphism analysis of the *TaDREB1* gene in wheat germplasm for the dissection of the drought resistance trait^56^.

The oil content is an important quality trait in peanut, but the genetic mechanism controlling peanut oil accumulation remains to be further studied. *GPAT9* genes have been confirmed to be key enzymes in TAG synthesis pathways in peanut and many other plants^18,19,21,57^. In our study, two *AhGPAT9* genes derived from the A- and B-genomes were obtained, allelic polymorphism analysis was conducted in 171 peanut germplasm, and primary correlation analysis was performed between alleles and seed oil content. Finally, we hypothesized the existence of high-oil or low-oil haplotypes or haplotype combinations. However, how the two *AhGPAT9* genes affect the seed oil accumulation needs to be further confirmed. Different hybrid combinations were assembled based on the high- or low-oil haplotypes of the two *AhGPAT9* genes. It is expected that the gene functions of *AhGPAT9A* and *AhGPAT9B* in regard to oil accumulation will be deeply analyzed and clarified in the future, and we further hope to create new germplasm with higher oil content via the hybrid polymerization of high-oil alleles of the *AhGPAT9A* and *AhGPAT9B* genes.

## Materials and methods

### Plant materials

A total of 171 cultivated peanut germplasm were used for the isolation and allele genotyping of *AhGPAT9*. All the peanut materials used in this study were planted from May to September in 2014, 2015, 2016 and 2017 in a test field at the Agricultural Experiment Station of Shandong Agricultural University (36.15°N, 117.15°E), Tai’an, China. The young leaves of each accession were collected and stored at -80 °C for DNA extraction, and the harvested seeds were used for oil content measurement.

### Detection of the oil content of peanut germplasm

The oil content was measured using a DA7250 Near Infrared Reflection (NIR) analyzer (Perten Instruments, Sweden), and the reference standard curve was also used in our previous study^38^. The heritability of oil content was calculated using the equation *h*_*B*_^2^ = V_G_ / (V_G_ + V_E_), where V_G_ and V_E_ represent genetic and environmental variation, and each term was extracted from the ANOVA results^58^. The mean value of each accession across four years was used in the statistical analysis, and one-way ANOVA was calculated by the least-significant difference (LSD) method^59^.

### DNA extraction, PCR amplification, and sequencing

Total genomic DNA was extracted from the leaves of each peanut accession using the hexadecyl trimethyl ammonium bromide (CTAB) method^60^. The DNA concentration and quality were estimated by using a NanoDrop2000 spectrophotometer (Thermo, USA) and 1% agarose gel electrophoresis in comparison with the relative migration and intensity of the standard 5 kb ladder (Takara Bio Inc, Japan). *AtGPAT9* (AT5G60620) from *Arabidopsis thaliana* was used as an information probe against the peanut database for expressed sequence tags (dbESTs) by employing BLAST analysis to retrieve homologous expressed sequence tags (ESTs). Ultimately, a single homologous sequence with an open reading frame (ORF) was established. We used the homologous sequence as a probe to search the website PeanutBase (https://peanutbase.org/). Two peanut *GPAT9* DNA sequences belonging to the wild species *A.duranensis* (AA) and *A.ipaensis* (BB) were retrieved and downloaded. We performed segment cloning, and ten primer pairs (S3 Table) were designed with Primer Premier 5.0 (https://www.premierbiosoft.com/) for amplifying each part of the *AhGPAT9* genes. The PCR mixture consisted of genomic DNA (50 ng), *TransStart® Taq* buffer (10 ×), a dNTP mixture (0.2 mM), primers (0.5 μM) and *TransStart® Taq* DNA Polymerase (1 U). PCR was performed with a thermocycling program of 95 °C for 5 min, 35 cycles of 95 °C for 30 s, 50–59 °C for 30 s and 72 °C for 1 min, and final step of 72 °C for 10 min. The PCR product was purified with the E.Z.N.A.TM Ploy Gel DNA Extraction Kit (Omega Bio-Tek, USA). DNA sequencing was performed on an ABI 3730XL automated sequencer following the manufacturer’s instructions (Applied Biosystems, Inc.) at Shanghai Personal Biotechnology (China).

### Allelic polymorphism analysis of *AhGPAT9*

Multiple sequence alignment analyses were carried out using DNAMAN software (https://www.lynnon.con/). The amino acid sequences were analyzed with the ExPASy web server (https://web.expasy.org/translate/), and the TMDs were predicted with TMHMM software (https://www.cbs.dtu.dk/services/TMHMM/). The protein spatial structures were determined by using I-TASSER^45–47^. DnaSP5.0 (https://www.ub.edu/dnasp/)^61^ was used for the assessment of genetic diversity including nucleotide diversity (π) and Tajima’s *D*^43^*.* Statistical analyses were based on the phenotypic data of the average oil content over four years. Variance analyses were performed with the SPSS System to determine phenotypic differences between the haplotypes both individually and in haplotype combinations, based on the analysis of variance (one-way ANOVA) according to the least-significant difference (LSD) test at the significance level of 5% (*P* ≤ 0.05).

### Expression analysis of *AhGPAT9* genes in different peanut tissues

Different peanut tissues were sampled, immediately frozen in liquid nitrogen, and stored at -80 °C for total RNA isolation, which was carried out by using a Quick RNA isolation kit (HuaYueYang Biotechnology, Beijing, China). First-strand cDNA synthesis was performed using the PrimeScript™ RT reagent kit with gDNA Eraser according to the manufacturer’s instructions (Takara Bio Company).

The expression analysis of *AhGPAT9* genes was performed by qRT-PCR using SYBR green PCR master mix in an ABI StepPlusone Fist Real-Time PCR system (ABI, USA). The primers for *GPAT9* genes were AF (5ʹ-TGTCAGTTCAGTGTTAGG-3ʹ), AR (5ʹ-TGGTGTGTCCAGAAGGTAGG-3ʹ), BF (5ʹ-GGTTCAATCGGACAGAGG-3ʹ) and BR (5ʹ-AAGTACCAAACATCACAC-3ʹ). The primers ACTF (5′-CAGCAGAGCGTGAAATCG-3′) and ACTR (5′-GGAAGAGCACCTCAGGACAA-3′) were utilized to amplify a 146 bp fragment of the reference gene *actin*^41,42^. The relative gene expression of *AhGPAT9* was calculated using the 2^-ΔΔCт^ methods^62^. Three replicates were used for each sample.

### Generation of transgenic peanut and seed oil content measurement

We generated two plant transformation constructs including one *AhGPAT9* overexpression vector (AhGPAT9-OE) and one anti-sense expression vector (AhGPAT9-AE). The *AhGPAT9A* sense coding sequence from Fenghua2 which was amplified from the cDNA clone with the forward primer BF1 (5ʹ-TTGCGGCCGCATGATGAGGAAGACCAATCCCAAGTC-3ʹ) containing a *BamH*I restriction site, and the reverse primer NR1 (5ʹ-CGCGGATCCTTACTTTTCTTCCAAGCGCCGGAGC-3ʹ), containing a *Not*I restriction site, was inserted between the CaMV 35S promoter and 35S terminator from the pGBVE plasmid to generate the AhGPAT9-OE constructs. A 1,128-bp fragment near the 3ʹ end of the *AhGPAT9A* coding region was amplified from the cDNA clone with the forward primer NF2 (5ʹ-TTGCGGCCGCATGATGAGGAAGACCAATCCCAAG-3ʹ), which contained a *Not*I restriction site and the reverse primer BR2 (5ʹ-CGCGGATCCTTACTTTTCTTCCAAGCGCCGGAGC-3ʹ), which contained a *BamH*I restriction site in Fenghua2 to produce the antisense copy. The *AhGPAT9* anti-sense fragment and pGBVE plasmid were digested with *Not*I and *BamH*I, and then cloned into the pGBVE vector under the control of the CaMV 35S promoter in the antisense orientation to generate an anti-sense expression vector. The *Bar* gene in the expression vectors confers glyphosate resistance and can be used as a selectable marker for screening transgenic plants. Then, different vectors were transformed into *Agrobacterium tumefaciens* strain LBA4404 via the freeze–thaw method. The recombinant bacteria were selected and used for the transformation of the FH2 cultivar using the *Agrobacterium*-mediated method and the leaflet regeneration system for peanut. The peanut seed leaflets of FH2 were exfoliated and cultured in medium containing 4.5 mg/L 6-BA and 0.7 mg/L NAA. The culture temperature was set to 27℃ ± 2℃, and culture was performed for 4 days under a cycle of 16 h light, 8 h darkness. Then, the cells were infected with the *Agrobacterium* suspension (OD = 0.65) containing the expression vector for 15 min and cultured for 4 days in the dark. The calluses were transferred to medium containing 4.5 mg/l 6-BA, 0.7 mg/L NAA and 500 mg/L Cef and cultured for 28 days, then transferred to medium containing 5 mg/L 6-BA for subsequent generation. The seedlings were cultured in medium containing 1 mg/L PPT for 1 month when the seedlings had grown to 3 cm, and the surviving peanut seedlings were propagated rapidly in MS medium.

To confirm the integration of the transgenic plants, the regenerated seedlings were cultured in medium containing 1 mg/L PPT for one month. The presence of the target constructs in the transgenic plants was confirmed by PCR in DNA isolated from the leaves of herbicide-resistant seedlings using the primers BarF (5ʹ-AAACCCACGTCATGCCAG-3ʹ) and BarR (5ʹ-CACCATCGTCAACCACTAC-3ʹ). The positive overexpression and anti-sense expression transformants were transferred to soil, and T_0_ transgenic seeds were harvested. T_0_ seeds were seeded as single seeds, and the presence of *AhGPAT9* in T_1_ was detected by PCR using the primers BarF and BarR. Independent overexpression and anti-sense expression transgenic peanut lines were selected in the T_2_ generation, and single plants from the T_2_ lines with a higher or lower oil content and *AhGPAT9* expression level than the wild-type FH2 were used to obtain the T_3_ transgenic lines. The seed oil content was determined using seeds from the T_1_, T_2_ and T_3_ generations of transgenic peanut. For every experiment, the transgenic experimental lines were grown in the same growth chamber at the same time as their corresponding negative control lines. To study the effect of *AhGPAT9* overexpression and antisense expression on peanut plant development, we collected the plant main stems and lateral branches of mature peanut plants and recorded the mature pod length, mature seed size and 100-seed weight from the transgenic lines in the T_3_ generation.

## Supplementary information

Supplementary file1

Supplementary file2

Supplementary file3

Supplementary file4
