# Supplementary material for: Characterization of glycerol-3-phosphate acyltransferase 9 (AhGPAT9) genes, their allelic polymorphism and association with oil content in peanut (Arachis hypogaea L.)
Source: Sci Rep. 2020 Sep 4;10:14648. doi: 10.1038/s41598-020-71578-7 (PMC7474056; doi:10.1038/s41598-020-71578-7)
Supplement: Supplementary file 2 — Supplementary file2 [file 41598_2020_71578_MOESM2_ESM.docx]

**Characterization of glycerol-3-phosphate acyltransferase 9 (*AhGPAT9*) genes, their allelic polymorphism and association with oil content in peanut (*Arachis hypogaea* L.)**

Yuying Lv ^1☯^, Xiurong Zhang^1☯^, Lu Luo^1^, Hui Yang^1^, Pinghua Li^1^, Kun Zhang^1^, Fengzhen Liu^1*^& Yongshan Wan^1*^

^1^ State Key Laboratory of Crop Biology, Shandong Key Laboratory of Crop Biology, College of Agronomy, Shandong Agricultural University, Tai’an, 271018 Shandong, China.

^☯^ Yuying Lv and Xiurong Zhang contributed equally to this work.

^*^Corresponding author: E-Mail: liufz@sdau.edu.cn (F. Liu), Tel.:+86 13854849601 & E-Mail: yswan@sdau.edu.cn (Y. Wan), Tel.: +86 13805386663

**
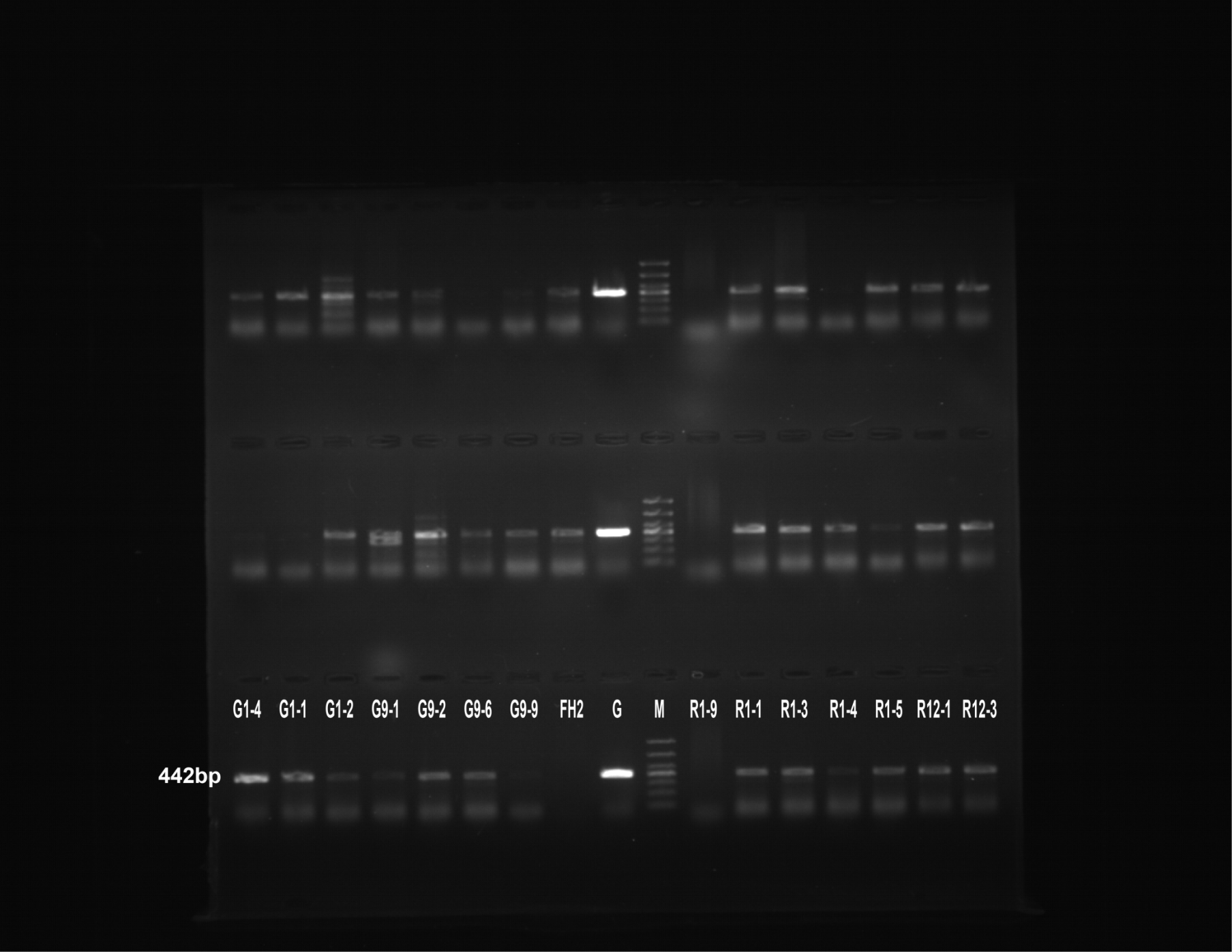
**

**This is the full-length gels for Fig 3B. G represented the recombinant plasmids, M was 1000 bp DNA Marker and FH2 represented wild-type (WT). G1-4, G1-1, G1-2, G9-2, G9-6, G9-1, and G9-6 were OE transgenic plants, and R1-9, R1-1, R1-3, R1-4, R1-5, R12-1 and R12-3 were AE transgenic plants.**

**Supporting information**

**Figures:**


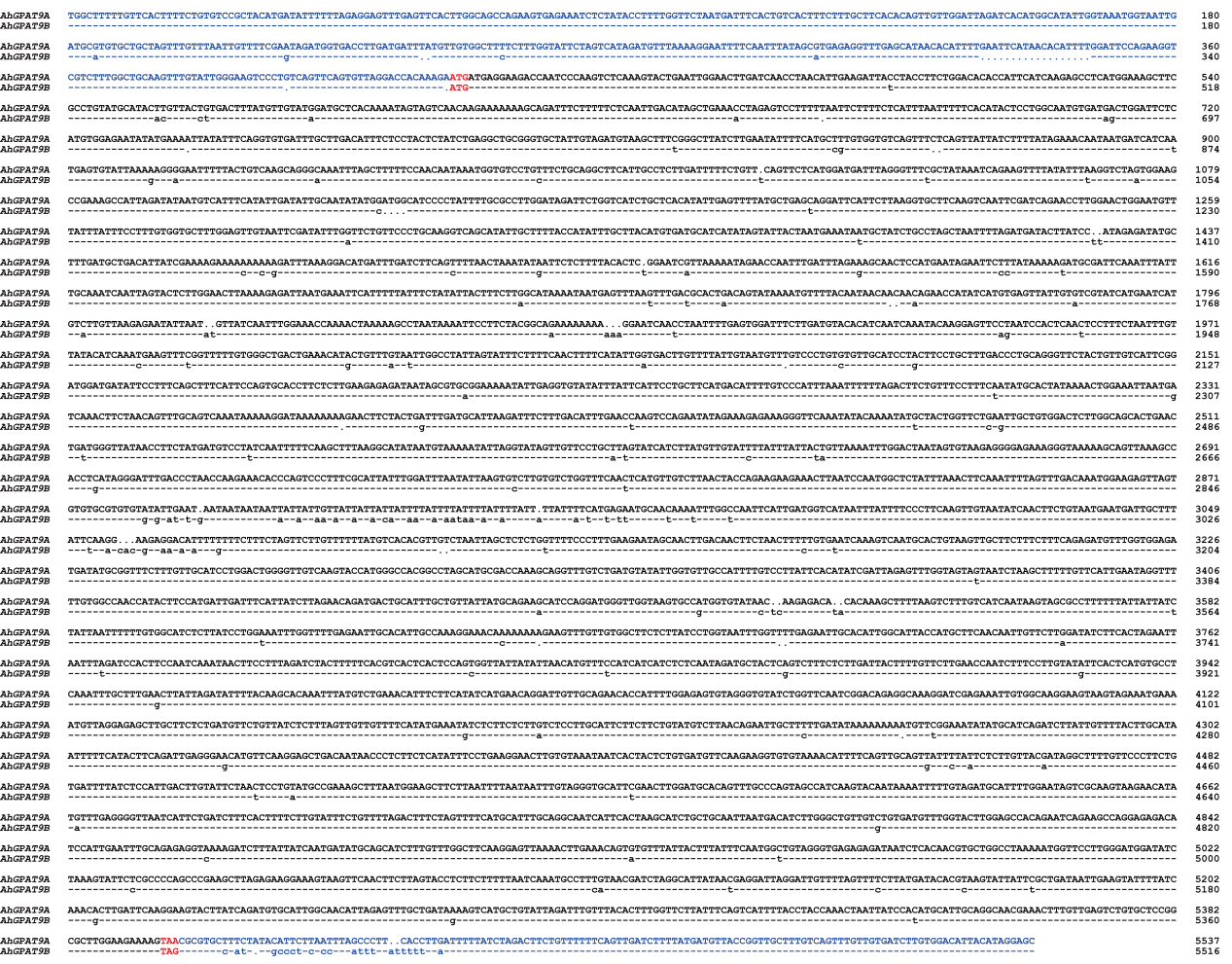


**S1 Figure Nucleic acid sequence alignment of *AhGPAT9A* and *AhGPAT9B*.** The red font represents the start codon ATG and the termination codon TAA or TAG. The blue font represented the 5'-UTR and 3'-UTR.


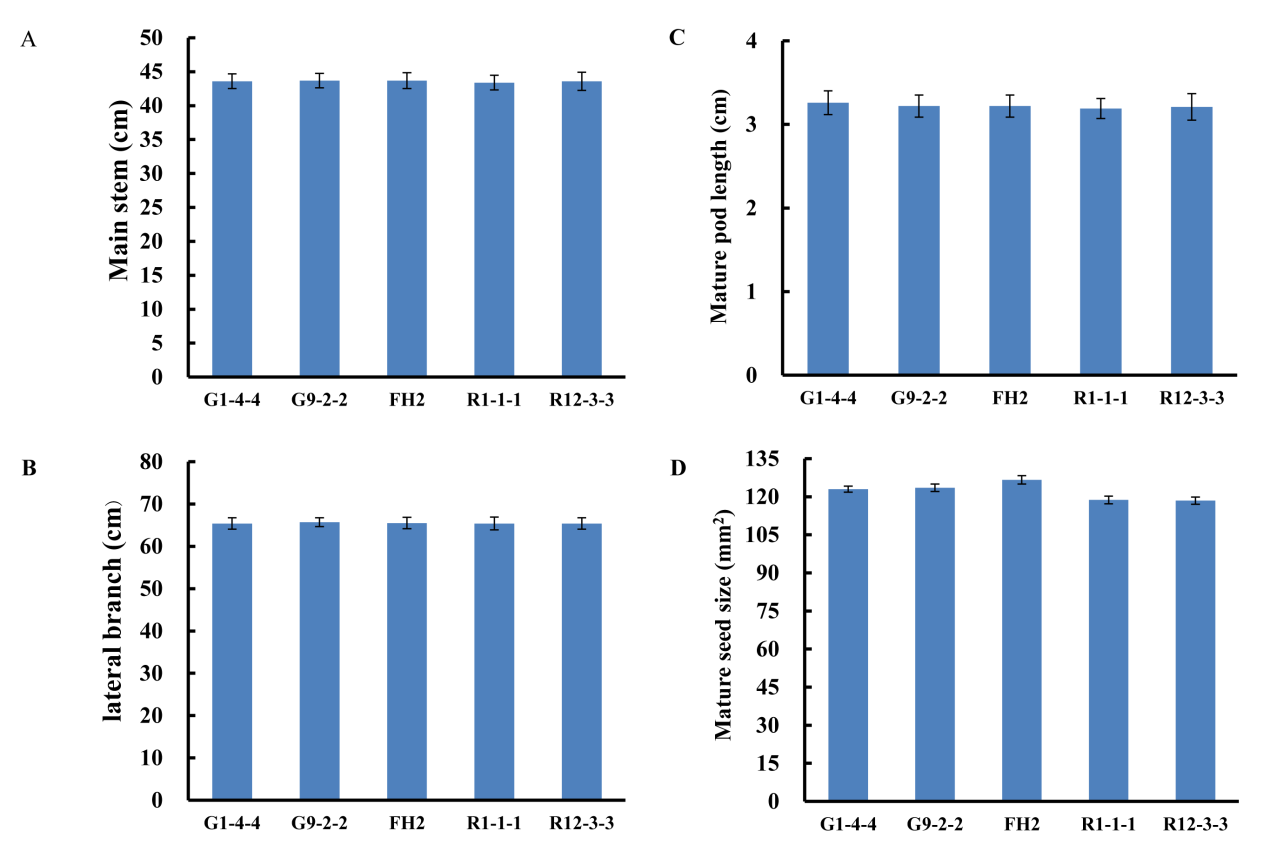


**S2 Figure Effect of *AhGPAT9* over-expression and anti-sense expression on peanut development.** A, Plant main stem of mature peanut. B, Plant lateral branch of mature peanut. C, Mature pod length. D, Mature seed size. Values are means ± SD of measurements on individual plants (n= 10).

**Tables:**

**S1 Table** **SNPs and InDels identified in the partial sequences of *AhGPAT9A* of peanut.**

**S2 Table SNPs and InDels identified in the partial sequences of *AhGPAT9B* of peanut.**

**S3 Table** **The primers and amplification sequence length of *AhGPAT9* genes.**
